# Escala predictiva de apendicitis para menores de 4 años: ¿Es posible aplicar la inteligencia artificial?

**DOI:** 10.31053/1853.0605.v81.n1.44316

**Published:** 2024-03-27

**Authors:** Dayhana Arango Cárdenas, Ximena Areiza Ocampo, Jorge Andrés Castrillón Lozano

**Affiliations:** 1 Universidad Cooperativa de Colombia Medellín Colombia; 2 Grupo de Investigación Infettare, Universidad Cooperativa de Colombia Medellín Colombia

**Keywords:** inteligencia artificial, apendicitis, pediatría, artificial intelligence, appendicitis, pediatric, inteligência artificial, apendicite, pediatria

## Abstract

La apendicitis aguda en la población pediátrica es una patología de presentación heterogénea que es diagnósticada actualmente mediante diversos criterios o escalas predictivas, que han demostrado no ser lo suficientemente precisas para ser estandarizadas, sin embargo, se han creado métodos que permitan establecer un diagnóstico más preciso, aspecto que ha sido proporcionado por la inteligencia artificial, la cual mediante diferentes algoritmos cuenta con la capacidad de arrojar cuál es el estado del paciente y la intervención más adecuada para este, disminuyendo así la tasa de intervenciones inecesarias y por ende posibles complicaciones relacionadas.

## Abstract (en)

Acute appendicitis in the pediatric population is a pathology of heterogeneous presentation that is currently diagnosed using various criteria or predictive scales, which have proven not to be sufficiently accurate to be standardized, however, methods have been created to establish a more accurate diagnosis, an aspect that has been provided by artificial intelligence, which through different algorithms has the ability to show the patient's condition and the most appropriate intervention for this, thus reducing the rate of unnecessary interventions and therefore possible related complications.

## Resumo

A apendicite aguda na população pediátrica é uma patologia de apresentação heterogénea que, atualmente, é diagnosticada através de vários critérios ou escalas preditivas, que se revelaram não suficientemente precisos para serem padronizados. No entanto, foram criados métodos para estabelecer um diagnóstico mais preciso, aspeto que tem sido proporcionado pela inteligência artificial, que através de diferentes algoritmos tem a capacidade de mostrar o estado do paciente e a intervenção mais adequada para o mesmo, reduzindo assim a taxa de intervenções desnecessárias e, consequentemente, as possíveis complicações relacionadas.

**CONCEPTOS CLAVE**

**Qué se sabe sobre el tema.**

La inteligencia artificial ha demostrado tener un gran impacto en la vanguardia actual del diagnóstico y han comenzado a surgir investigaciones enfocadas hacia la apendicitis aguda en pacientes pediátricos y en su mayoría han evaluado la sensibilidad y especificidad de este en comparación con la de los diversos criterios que han sido creados y son aplicados en la práctica médica.

**Qué aporta este trabajo.**

Un método alterno, novedoso, que permite realizar un diagnóstico y enfoque más preciso y acertado de la apendicitis aguda, disminuyendo así la tasa de intervenciones quirúrgicas innecesarias, y dando pie a la posibilidad de ampliar las investigaciones en esta área.

**Divulgación**

La inteligencia artificial es la manera en que, mediante softwares de computadora se han logrado facilitar muchas tareas de la vida cotidiana. La medicina no ha sido ajena a estos novedosos métodos, hoy en día se han implementado para realizar diagnósticos y clasificaciones de algunas enfermedades, entre muchas otras tareas. De esta manera, la apendicitis aguda en niños, es una condición que varía mucho entre paciente y paciente, pudiendo ocasionar que existan errores en el diagnóstico y para esto, la inteligencia artificial nos ha permitido crear softwares y programas computacionales que ayuden al personal médico a realizar un correcto enfoque de esta enfermedad tan común.

Hemos leído con interés el trabajo de Rassi et al. ^1^ titulado: "Escala predictiva de apendicitis para menores de 4 años" que tuvo por objetivo establecer la capacidad y precisión diagnóstica de una escala fundamentada en variables medidas en pediatría ambulatoria y de urgencias.

Se han creado una gran variedad de puntuaciones que permiten diagnosticar acertadamente la apendicitis aguda en niños, sin embargo, ninguna ha sido exactamente precisa para esto. Similar al enfoque de los autores, Aydoğdu et al. ^2^ desarrollaron un sistema de puntuación basado en parámetros hematológicos, ajustados por edad y sexo, en el que se analizaron 946 pacientes y determinaron una sensibilidad del 94% y una especificidad del 86%, no obstante, recalcaron que dichos datos varían según aspectos demográficos del paciente, por lo que se requiere tenerlos en cuenta al momento de evaluar los resultados.

Los marcadores sanguíneos inflamatorios y diversos métodos de imagen como la ecografía tienen una precisión limitada, ya que deben ser interpretados por un experto, limitando la certeza diagnóstica por la baja fiabilidad inter-observador. Hoy en día, con el advenimiento de las nuevas tecnologías, la inteligencia artificial (IA) plantea un método basado en programas automatizados o softwares que permiten tomar decisiones en función de los datos añadidos, demostrando ser de gran ventaja para el diagnóstico certero de diversas patologías, entre ellas, la apendicitis aguda. Reismann et al. ^3^ , crearon un algoritmo de aprendizaje mediante IA como modelo de predicción para la apendicitis basado en biomarcadores de relevancia, permitiendo detectar y diferenciar una apendicitis complicada, dicha estrategia arrojó una sensibilidad del 100% y una especificidad del 97%. Por ende, concluyeron que es un método que, a pesar de requerir mejoras significativas, en comparación con otros, tiene un valor significativo relevante para descartar apendicitis, por su alta sensibilidad y especificidad.

La IA resulta de un enfoque novedoso en la actualidad para diagnosticar acertadamente y prevenir cirugías innecesarias en población pediátrica, Aydin et al. ^4^ , desarrollaron un algoritmo con enfoque de aprendizaje automático que incluía datos demográficos y de laboratorio y mediante el análisis de 7244 pacientes con una edad media de 6,84 ± 5,31 años, arrojó una sensibilidad del 93,5% y una especificidad del 96,5%, permitiendo distinguir una apendicitis aguda de una complicada, por tanto, se concluyó que es un método potencial en rápido progreso, que brindará mejoras en las estadísticas diagnósticas y de morbimortalidad, dado que es un modelo que puede automatizarse y es altamente preciso, aspecto que irá aumentando una vez se incluyan más datos de pacientes en el algoritmo del software.

La apendicitis es una patología de presentación heterogénea con diagnóstico esencialmente clínico, apoyado por datos de laboratorio e imágenes, a pesar de esto, se ha demostrado que la IA ofrece mayor precisión en dicho aspecto, debido a que sus algoritmos de aprendizaje pueden ser entrenados, para que entre más sea el flujo de datos ingresado a sus softwares, mayor precisión puedan obtener. Marcinkevics et al. ^5^ , realizó un algoritmo de aprendizaje automático en el que incluyó datos como puntuación de Alvarado, diámetro del apéndice, temperatura, recuento de leucocitos, porcentaje de neutrófilos, marcadores inflamatorios y presencia peritonitis/defensa abdominal, con el objetivo de diagnosticar acertadamente, guiar el manejo y estratificar la gravedad, ofreciendo beneficios con respecto a la detección temprana y el seguimiento de complicaciones, arrojando una sensibilidad del 100% y una especificidad del 95%.

En conclusión, se han propuesto diversidad de escalas predictoras para el diagnóstico de apendicitis aguda en la población pediátrica, utilizando distintos parámetros clínicos, paraclínicos e imagenológicos y aplicándolas en muestras de distintas características. En el estudio de Rassi et al. ^1^ , hallaron una sensibilidad del 95.1% y una especificidad del 90%, pudiendo predecir adecuadamente el riesgo para apendicitis aguda pediátrica. Se vale recalcar que, los modelos basados en inteligencia artificial creados para este fin, han demostrado valores predictivos, sensibilidad y especificidad superiores a las escalas previamente propuestas y es menester que las investigaciones que actualmente están enfocadas a desarrollar escalas de predicción, puedan analizar la posibilidad de incluir la inteligencia artificial en sus propuestas de escalas diagnósticas, garantizando un enfoque multidisciplinar que pueda prevenir de manera más precisa los diagnósticos erróneos y las cirugías innecesarias.

## References

[1] Rassi R, Muse F, Cuestas E (2023). Escala predictiva de apendicitis para menores de 4 años. Rev Fac Cien Med Univ Nac Cordoba.

[2] Aydoğdu B, Azizoğlu M, Arslan S, Aydoğdu G, Basuguy E, Salık F, Ökten M, Hanifi-Okur M (2023). Nuevo sistema de calificación diagnóstica para apendicitis pediátrica basado en parámetros hematológicos ajustados por edad y sexo. Gac Med Mex.

[3] Reismann J, Romualdi A, Kiss N, Minderjahn MI, Kallarackal J, Schad M, Reismann M (2019). Diagnosis and classification of pediatric acute appendicitis by artificial intelligence methods: An investigator-independent approach. PLoS One.

[4] Aydin E, Türkmen İU, Namli G, Öztürk Ç, Esen AB, Eray YN, Eroğlu E, Akova F (2020). A novel and simple machine learning algorithm for preoperative diagnosis of acute appendicitis in children. Pediatr Surg Int.

[5] Marcinkevics R, Reis Wolfertstetter P, Wellmann S, Knorr C, Vogt JE (2021). Using Machine Learning to Predict the Diagnosis, Management and Severity of Pediatric Appendicitis. Front Pediatr.

